# Atypical Findings of Shwachman-Diamond Syndrome in Early Infancy: A Diagnostic Challenge

**DOI:** 10.1097/PG9.0000000000000165

**Published:** 2022-01-24

**Authors:** Concetta Marsico, Andrea Scozzarella, Maria Grazia Capretti, Filomena Carfagnini, Elena Facchini, Santo Arcuri, Arianna Aceti

**Affiliations:** From the * Department of Medical and Surgical Sciences, University of Bologna, Bologna, Italy; † Neonatal Intensive Care Unit, IRCCS Azienda Ospedaliero-Universitaria di Bologna, Bologna, Italy; ‡ Pediatric Radiology, IRCCS Azienda Ospedaliero-Universitaria di Bologna, Bologna, Italy; § Pediatric Oncology and Hematology Unit, IRCCS Azienda Ospedaliero-Universitaria di Bologna, Bologna, Italy.

**Keywords:** Shwachman-Diamond syndrome, pancreatic insufficiency, low birth weight, anemia

## Abstract

Shwachman-Diamond syndrome (SDS) is a rare autosomal recessive disorder characterized by hematological abnormalities, exocrine pancreatic insufficiency, and skeletal dysplasia. We describe a 2-month-old girl with intrauterine and extrauterine growth restriction who presented with an isolated severe anemia requiring red blood cell transfusion, without gastrointestinal symptoms, history of infection, or congenital abnormalities. An abdominal ultrasound revealed a reduced pancreatic thickness and abnormal echogenicity without fat infiltration, further confirmed by MRI. Because of this peculiar pancreatic appearance, pancreatic function was investigated and revealed exocrine insufficiency. Genetic testing confirmed SDS diagnosis. The typical clinical, laboratory, and imaging features of SDS are often lacking in the first months of life, and this may delay diagnosis. In early infancy, low birth weight and lack of catch-up growth, isolated hematological abnormalities other than neutropenia and atypical pancreatic imaging may lead to SDS diagnosis even when the most common diagnostic criteria are not fulfilled.

## INTRODUCTION

Shwachman-Diamond syndrome (SDS) is a rare autosomal recessive disorder with an estimated incidence of 1/76,000 (1). Main SDS features are bone marrow dysfunction, typically characterized by neutropenia, exocrine pancreatic insufficiency, and congenital anomalies, but since the advent of genetic testing an unexpectedly broad range of phenotypes has become clear (1–3).

We describe an atypical, early-onset SDS presentation, where diagnosis was misled by unusual clinical characteristics and confounding laboratory and imaging findings.

## CASE REPORT

A female infant was delivered vaginally at 37+6 weeks of gestational age after an uneventful pregnancy from nonconsanguineous parents. The infant was small-for-gestational-age (SGA): birth weight (BW) was 2090 g (–3.77 SD), length 47 cm (–1.15 SD), and occipital-frontal circumference (OFC) 32 cm (–1.59 SD).

At 2 months of age, she presented pale and tachycardic at a routine clinical evaluation. Blood tests documented severe anemia (hemoglobin [Hb] 6.9 g/dL), and the infant was admitted to the Neonatal Unit, where a red blood cells (RBC) transfusion was performed.

Upon admission, she was in good clinical conditions, without dysmorphic features, but showing further extrauterine growth retardation (weight 2840 g [–4.19 SD]).

Blood tests documented normocytic anemia with low reticulocytes count, normal platelet, and white blood cells counts, with an absolute neutrophil count of 1060/mmc. Peripheral blood smear, Coombs test, hemolysis indexes, urine analysis, fecal occult blood, blood real-time PCR for Parvovirus B-19, enteroviruses and herpetic viruses, serology for *T.pallidum* and iron status assessment were normal.

**FIGURE 1.**
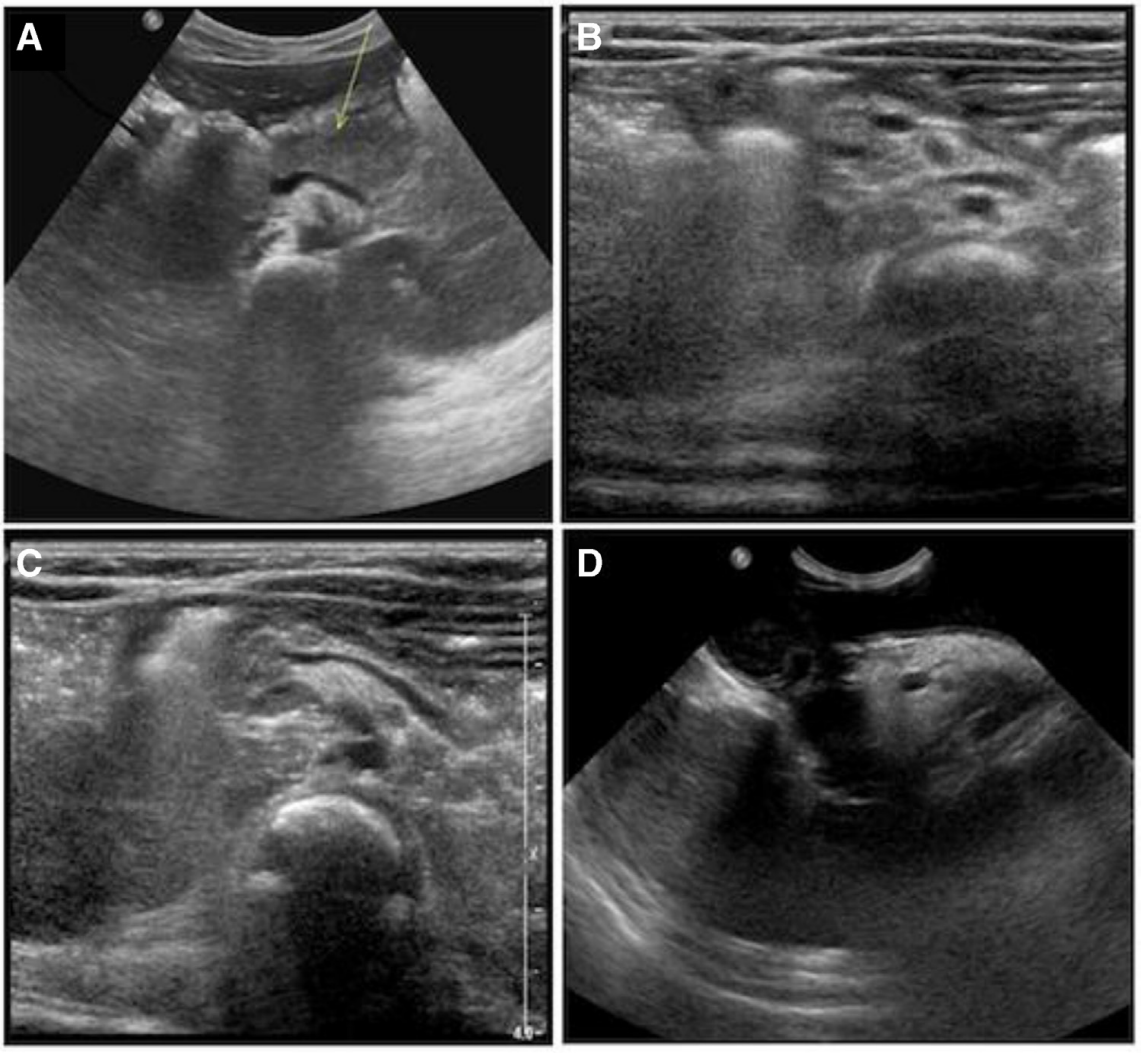
US images. (A) Normal pancreas size and echogenicity of a healthy 2-month-old infant. (B, C) Reduced thickness and abnormal echogenicity of the pancreas at 2 months of age in the present case. (D) Hyperechoic pancreas with increased size compared with the previous US appearance at 9 months of age. US = ultrasound.

**FIGURE 2.**
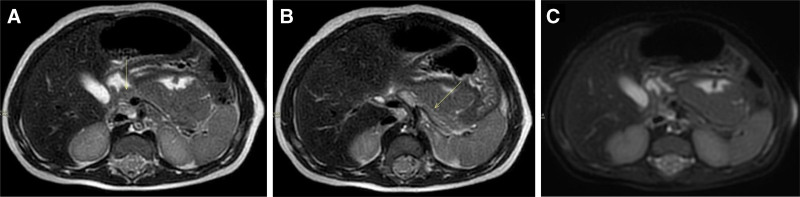
Abdominal MRI sequences. (A, B) Axial T2 weighted TSE MV sequences showing reduced thickness of the pancreas, particularly affecting the pancreas body and tail. (C) Axial T2 weighted TSE MV sequences with selective fat suppression (Spectral Presaturation with Inversion Recovery) at the same level showing no lipomatosis. MRI = magnetic resonance imaging; MV = multivane; TSE = Turbo-Spin-Echo.

An abdominal ultrasound (US) revealed a small-sized pancreas, with reduced thickness, particularly of the body and tail, abnormal echogenicity, and a “damaged” appearance (Figure 1). An abdominal MRI, including T2 weighted Turbo-Spin-Echo Multi-Vane sequences with selective fat suppression, confirmed the reduction of the pancreas parenchyma, without any fat infiltration or calcification (Figure 2).

Pancreatic function was explored: fecal elastase values were within the range of severe pancreatic insufficiency (<15 μg/g wet stool, normal >200 μg/g).

In the suspicion of an atypical form of cystic fibrosis (CF), a sweat test was undertaken and was normal (41 mmol/L, normal < 50 mmol/L) and the genetic test for CF transmembrane conductance regulator (*CFTR*) gene mutations was required.

Blood exams showed a slow improvement of the Hb value, persistent reticulocytopenia, and in one sample neutropenia was documented (neutrophil count range 500–1937/mmc). In the suspicion of SDS, molecular analysis of *SBDS* gene was required.

A radiograph of the thoracic cage was performed and revealed the presence of 11 ribs bilaterally, without further abnormalities. Prothrombin time and activated partial thromboplastin time, serum calcium, phosphorus, and alkaline phosphatase were in the normal range for age.

The infant was discharged after 24 days, with a weight of 3354 g (–4.15 SD), in good clinical conditions, without gastrointestinal (GI) symptoms but on vitamins and trace elements supplementation.

Genetic analyses revealed no causative mutations in the *CFTR* gene. The patient was instead found to be homozygous for the exon 2 mutation c.258+2 T > C in the *SBDS* gene, causative of SDS.

## DISCUSSION

SDS early diagnosis may be challenging because the disease typical features are often lacking in the first months of life. Nonetheless, a high index of suspicion when only subtle and nonspecific but consistent findings are present may lead to a genetic diagnosis even in early infancy.

To date, only few cases of SDS presentation and molecular diagnosis in the first months of life have been documented (Table 1) (4–7). More commonly, patients diagnosed with SDS in older ages are retrospectively recognized with signs consistent with SDS since birth, with a median age at diagnosis between 1 and 3.5 years (1,2). The five youngest described patients share several clinical features, including SGA status. The most common hematological abnormality is pancytopenia with persistent neutropenia, leading to recurrent infectious complications (5–7). Anemia is consistently described, even though the Hb value is reported only in two patients (5.9 g/dL and 6.8 g/dL, respectively) (4,7). Pancreatic insufficiency, as revealed by low fecal elastase or poor postnatal growth is also universally present, but most commonly without GI symptoms (4–7).

**TABLE 1.** Summary of case reports of SDS early presentation (<3 months)

	Saito-Benz et al (2015)	Schaballie et al (2013)		Andolina et al (2013)	Black et al (2008)	Present case (2020)
Gender	M	F	M	M	F	F
Age at diagnosis	<1 month	2 months	7 days	3 months	<3 month	2 months
Small for gestational age	+	+	+	N/A	+	+
Birth weight (percentile)	3°	<3°	3°–5°	N/A	N/A	<3°
Birth length (percentile)	<3°	N/A	N/A	N/A	N/A	>10°
Birth occipital-frontal circumference (percentile)	50°	N/A	N/A	N/A	N/A	3°–10°
Respiratory distress at birth	+	N/A	N/A	N/A	+	–
Poor postnatal growth	+	+	+	–	–	+
Anemia	+	+	+	+	+	+
Neutropenia*	+/P	+/P	+/P	+/P	+/P	+/I
Thrombocytopenia	+	+	+	–	+	–
Pancreatic functionality’s test†	N/A	+	+	+	+	+
Gastrointestinal symptoms‡	+	N/A	N/A	–	N/A	–
Skeletal dysplasia	+	–	+	–	+	–
Recurrent infections/sepsis	+	+	+	+	N/A	–
Abnormal abdominal ultrasound	–	N/A	N/A	N/A	N/A	+

*P = persistent, I = intermittent.

†At least one of trypsinogen, amylase, fecal elastase that shows a pancreatic insufficiency.

‡Steatorrhea, liquid stool, deficiency of vitamins.

N/A = not available.

In line with these data, our patient was SGA and showed no catch-up growth in the first months of life. The main symptom leading to hospitalization was a severe anemia, which seems to be common even though rarely isolated. A severe anemia requiring transfusion has been reported in 8% of subjects at presentation but always in association with neutropenia or pancytopenia (1).

In healthy pediatric patients, abdominal US is considered the first noninvasive imaging technique to assess pancreatic size and structure, which is usually well marginated and homogeneous, with an echogenicity equal or lower than the liver (Figure 1). MRI, when performed with specific sequences such as fat suppression sequences, offers higher sensitivity and specificity for detecting the typical fat replacement as compared with US (8). It has been suggested that patients with confirmed *SBDS* gene mutation exhibit a characteristic MRI pattern of pancreatic fatty replacement, while patients without *SBDS* mutations usually have a normal signal intensity despite confirmed exocrine pancreatic dysfunction (9). Data from the North American SDS registry reported pancreatic US studies in 17 patients: 14/17 (82%) showed pancreatic lipomatosis; one patient had an initially normal pancreatic US, which evolved into lipomatosis after 3 years; two patient had no pancreatic lipomatosis; and one of them had a small pancreas (1), which has been reported also in other 3 young patients (<1 year) with clinically diagnosed SDS (2).

In the present case, US and MRI pancreatic features were at first considered as not consistent with SDS. It is likely that these unusual findings were related to the young age. Indeed, the US performed at 9 months of age showed a larger and more hyperechoic pancreas (Figure 1).

SDS still carries a high risk of misdiagnosis, especially in the youngest patients, where the typical clinical, laboratory, and imaging features are often lacking. Common clinical findings are low BW and lack of catch-up growth, without specific GI symptoms. Even if hematological abnormalities are always present, only one bone marrow lineage, not necessarily the neutrophil count, may be affected; an unexplained severe anemia may also be present. Imaging findings may not be suggestive of SDS, as the typical lipomatosis may be lacking. The only feature which is constantly present is the laboratory evidence of exocrine pancreatic insufficiency, which should be looked for even when clinical presentation fails to meet the most common diagnostic criteria.

## ACKNOWLEDGMENTS

Informed consent was obtained from the infant’s parents for the publication of this case report.
